# Supplementary material for: Pain-causing stinging nettle toxins target TMEM233 to modulate NaV1.7 function
Source: Nat Commun. 2023 Apr 28;14:2442. doi: 10.1038/s41467-023-37963-2 (PMC10147923; doi:10.1038/s41467-023-37963-2)
Supplement: Supplementary file 1 — Supplementary Information [file 41467_2023_37963_MOESM1_ESM.pdf]

Supplementary information

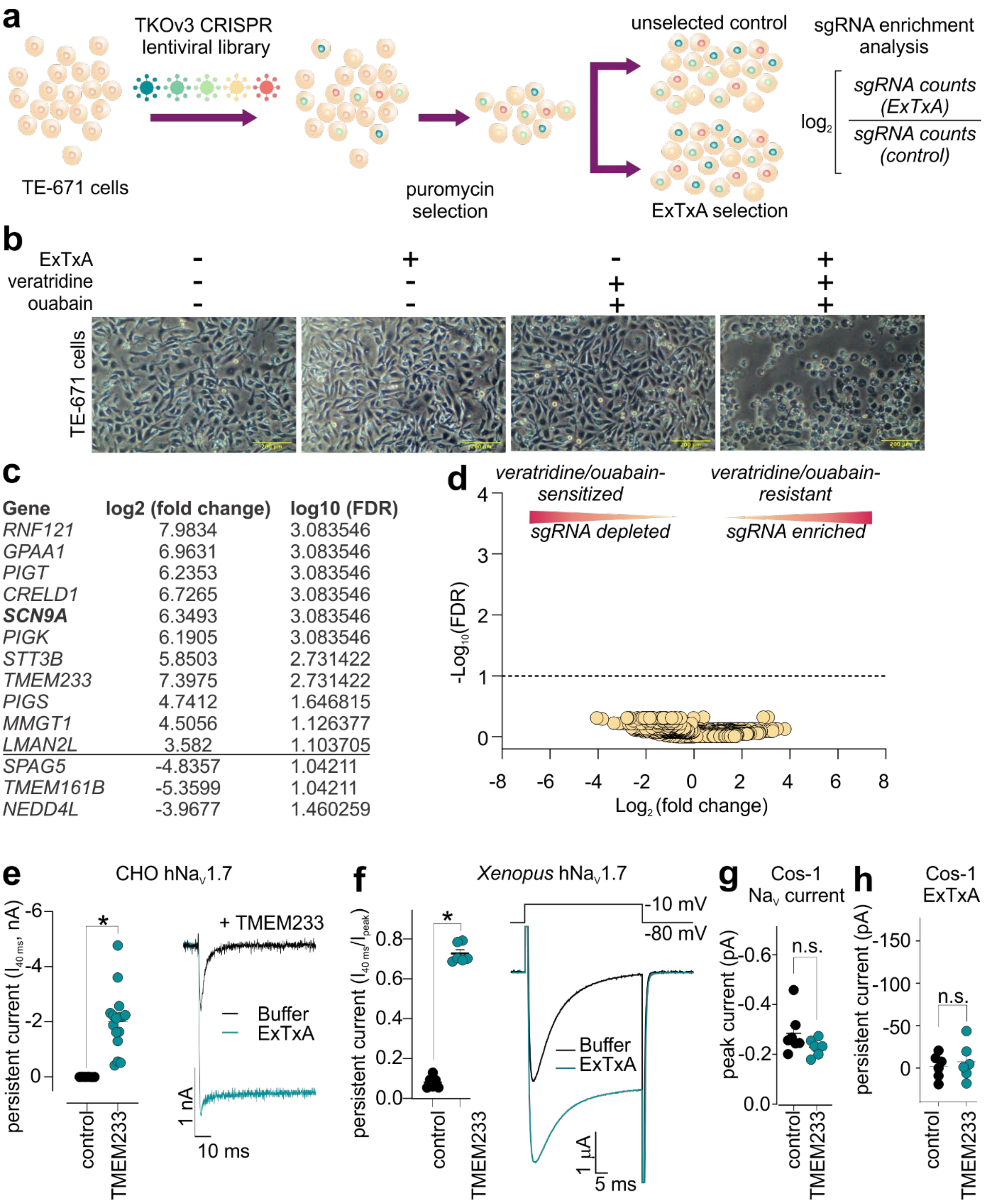

Supplementary Fig. S1. A CRISPR knockdown screen in TE-671 cells identifies TMEM233 as the molecular target of ExTxA.

a) *Schematic illustrating the LentiCRISPR-Cas9 TKOv3 screen used to identify the genes required for ExTxA-induced cytotoxicity.* ***b)*** *Representative light microscope images showing*

the effect of ExTxA (1 μM), veratridine (5 μM) and ouabain (20 nM) on viability of TE-671 cells after 24 h. Scale bars, 200 μm. ***c)*** *Gene names, log 2 (fold change) and log10 FDR (false discovery rate) of hits from ExTxA-selection in TE-671 cells.* ***d)*** *LentiCRISPR-Cas9 TKOv3 screen showing no depletion or enrichment of sgRNAs in control (untreated) and veratridine/ouabain-treated (veratridine, 5 μM; ouabain, 20 nM) TE-671 cells.* ***e)*** *ExTxA (1 μM)-induced persistent current ($I_{40\ ms}$, nA) in CHO cells expressing hNa$_V$1.7 alone (control) or co-expressing hNa$_V$1.7 and TMEM233 (TMEM233). Right; representative current traces; depolarization to -20 mV from -90 mV holding potential.* ***f)*** *ExTxA (1 μM)-induced normalized persistent current ($I_{40\ ms}/I_{peak}$) in* Xenopus *oocytes expressing hNa$_V$1.7 alone (control) or co-expressing hNa$_V$1.7 and TMEM233 (TMEM233). Right; representative current traces; depolarization to -10 mV from -80 mV holding potential.* ***g)*** *Depolarization-induced (holding potential -90 mV, 50 ms step to -20 mV) peak current (pA) in untransfected Cos-1 cells (control) and Cos-1 cells transfected with TMEM233 only (TMEM233).* ***h)*** *ExTxA (1 μM)-induced persistent current (40 ms; pA) in untransfected Cos-1 cells (control) and Cos-1 cells transfected with TMEM233 only (TMEM233) during depolarization to -20 mV from a holding potential of -90 mV. Data are shown as mean ± SEM; n.s., not significant; *, $p < 0.05$. n values and statistical information are detailed in Supplementary data Table 1. Source data are provided as a Source Data file.*

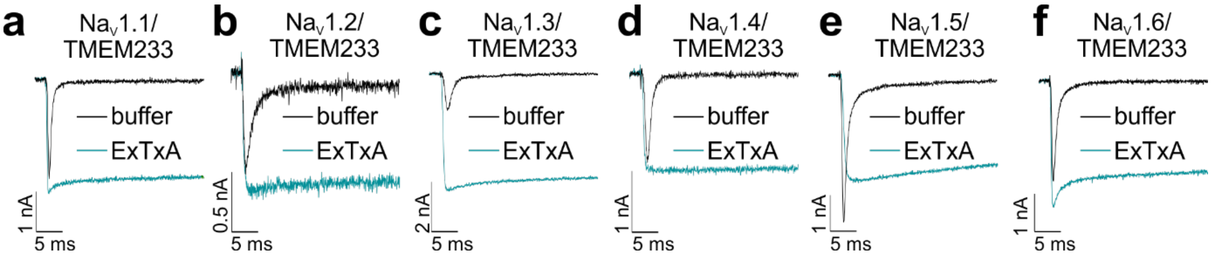

Supplementary Fig. S2. Co-expression of TMEM233 confers ExTxA sensitivity to Na$_V$1.1-1.6.

Representative current traces showing ExTxA-induced persistent currents in HEK293 cells expressing ***a)*** *Na$_V$1.1 + TMEM233,* ***b)*** *Na$_V$1.2 + TMEM233,* ***c)*** *Na$_V$1.3 + TMEM233,* ***d)*** *Na$_V$1.4 + TMEM233,* ***e)*** *Na$_V$1.5 + TMEM233, and* ***f)*** *Na$_V$1.6 + TMEM233.*

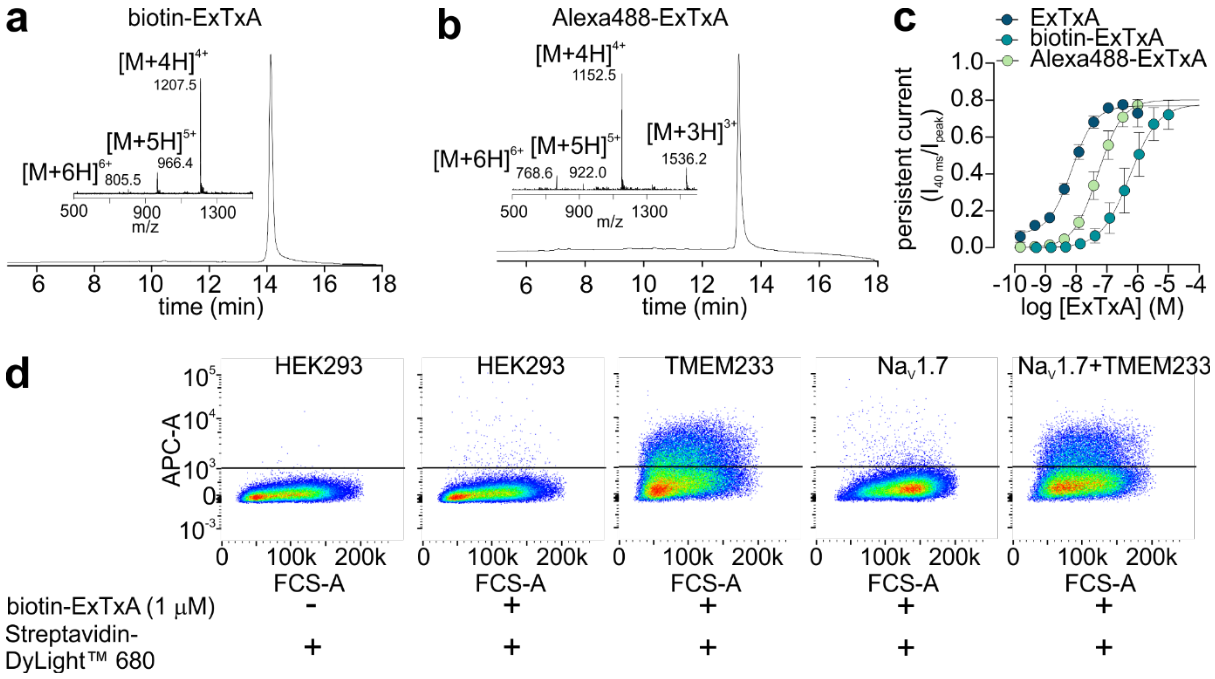

Supplementary Fig. S3. Biotin- and Alexa488-labelled ExTxA as probes to determine TMEM233 binding.

UPLC and mass spectrometry profile of ***a)*** *N-terminally biotinylated ExTxA and* ***b)*** *N-terminally Alexa488-tagged ExTxA.* ***c)*** *Concentration-response of native, biotinylated and Alexa488-tagged ExTxA in HEK293-Na$_V$1.7 cells co-transfected with TMEM233.* ***d)*** *Representative flow cytometry analysis of untransfected (HEK293) and TMEM233-transfected HEK293 cells, as well as HEK293 cells expressing hNa$_V$1.7 alone (Na$_V$1.7) or co-expressing hNa$_V$1.7 and TMEM233 (Na$_V$1.7+TMEM233) stained with biotinylated ExTxA and DyLight^{TM}-680-conjugated streptavidin. Solid line indicates cut-off for signal-positive cells. Data in c) are shown as mean ± SEM. n values and statistical information are detailed in Supplementary data Table 1.*

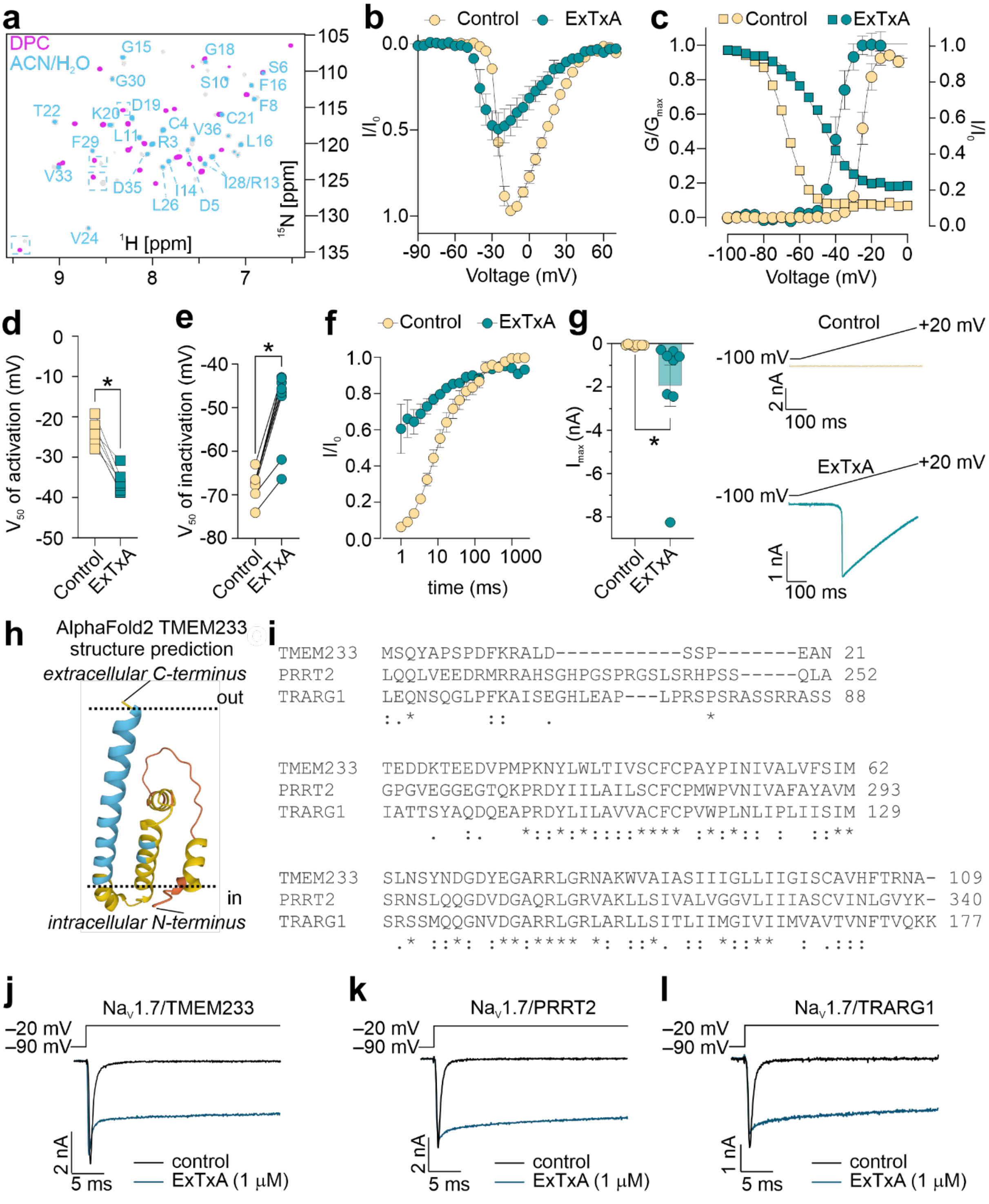

Supplementary Fig. S4. Effects of ExTxA on Na$_V$1.7 co-expressed with the dispanins.

a) *^{1}H-^{15}N transverse relaxation optimized spectroscopy (TROSY) spectra of ^{15}N labelled recombinant ExTxA in acetonitrile/H$_2$O and dodecylphosphocholine micelles (DPC) showing distinctly different chemical shifts in a lipid membrane-like environment.* ***b-g)*** *Electrophysiological characterization of ExTxA effects on Na$_V$1.7 co-expressed with*

*TMEM233. **b)** Current-voltage (IV) relationship, **c)** conductance-voltage (GV, circles) and steady-state inactivation (squares) curves, **d)** V_{50} of activation and **e)** V_{50} of steady-state fast inactivation as well as **f)** time-dependence of recovery from fast inactivation and **g)** (left) peak ramp currents and (right) representative ramp (0.2 mV/s) current traces from HEK293 cells co-expressing $Na_V1.7$ and TMEM233 after the addition of 0.1% BSA (yellow, control) and ExTxA (teal, 1 µM in 0.1% BSA). **h)** AlphaFold2 TMEM233 (UniProt ID B4DJY2) structure prediction. Dotted lines indicate approximate locations of plasma membrane. **i)** Sequence alignment of the human dispanins TMEM233 (DSPB2; UniProt ID B4DJY2), PRRT2 (DSPB3; UniProt ID Q7Z6L0), and TRARG1 (DSPB1; UniProt ID Q8IXB3). The large N-terminal domains of PRRT2 and TRARG1 are truncated for clarity. **j)** Representative current traces from HEK293-$Na_V1.7$ cells co-transfected with TMEM233, **k)** PRRT2 or **l)** TRARG1 showing ExTxA (1 µM)-induced inhibition of inactivation. Shown is a 50 ms depolarization to -20 mV from a holding potential of-90 mV. Data are shown as mean ± SEM; *, $p < 0.05$. n values and statistical information are detailed in Supplementary data Table 1.*

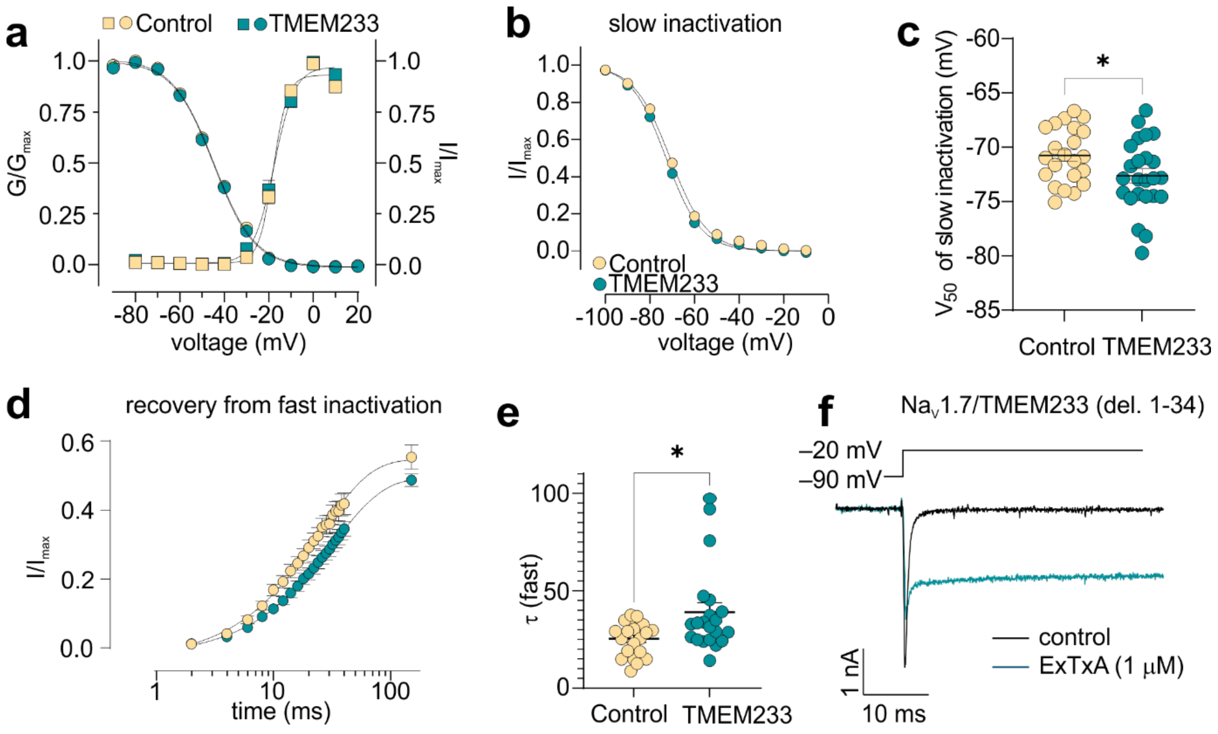

Supplementary Fig. S5. Effect of TMEM233 co-expression on Na$_V$1.7 expressed in* Xenopus *oocytes.

***a)** Superimposed conductance–voltage (GV, squares) and steady-state fast inactivation (circles) curves of* Xenopus *oocytes expressing hNa$_V$1.7 alone (control, yellow) or co-expressing hNa$_V$1.7 and TMEM233-transfected (TMEM233, teal). **b)** Voltage-dependence and **c)** V_{50} of slow inactivation of hNa$_V$1.7 alone (control, yellow) or hNa$_V$1.7 co-expressed with TMEM233 (TMEM233) in* Xenopus *oocytes. **d)** Time-dependence and **e)** time constant τ of recovery from fast inactivation of hNa$_V$1.7 alone (control, yellow) or hNa$_V$1.7 co-expressed with TMEM233 (TMEM233) in* Xenopus *oocytes. **f)** Representative current trace of ExTxA (1 μM)-induced persistent current in HEK293-hNa$_V$1.7 cells co-expressing N-terminally truncated TMEM233 (del. 1-34). Data are shown as mean ± SEM; *, $p < 0.05$. n values and statistical information are detailed in Supplementary data Table 1.*

Supplementary Table 1.

Figure	Parameter (units)	Group	mean ± SEM	n	Statistical test
**Fig. 1a,b**	DRG total persistent Na_V current ($I_{40\ ms}/I_{peak}$)	Control	0.027 ± 0.01	6	* $p< 0.05$ by repeated measures one-way ANOVA with Tukey's multiple comparison test
		ExTxA (100 nM)	0.513 ± 0.10		
		ExTxA (100 nM) + TTX (1 µM)	0.001 ± 0.004		
**Fig. 1c,d**	DRG $Na_V1.8$ Δ persistent current ($I_{40\ ms}/I_{peak}$):	Control	0.009 ± 0.005	13	$p = 0.024$ by two-sided Mann-Whitney test
		ExTxA (100 nM)	0.050 ± 0.015	13	
**Fig. 1e,f**	DRG $Na_V1.9$ Δ persistent current ($I_{90\ ms}/I_{peak}$):	Control	-0.034 ± 0.015	9	$p = 0.447$ by two-sided t-test
		ExTxA (1 µM)	-0.057 ± 0.028	7	
**Fig. 1g,h**	hiPSC-derived sensory neurons persistent current ($I_{40\ ms}/I_{peak}$):	Buffer control (0.1% BSA)	0.025 ± 0.009	5	*$p = 0.0002$ (control vs ExTxA); #, $p = 0.0123$ (ExTxA vs ExTxA + Pn3a) by Repeated measures one-way ANOVA with Tukey's multiple comparison test
		ExTxA (100 nM)	0.151 ± 0.024		
		ExTxA (100 nM) + Pn3a (100 nM)	0.069 ± 0.012		
		ExTxA (100 nM) + Pn3a (100 nM) + TTX (1 µM)	0.003 ± 0.001		
**Fig. 1i,j**	TE-671 persistent current ($I_{40\ ms}/I_{peak}$)	Buffer control	0.021 ± 0.009	8	*,#$p < 0.001$ by Repeated measures one-way ANOVA with Tukey's multiple comparison test
		ExTxA (100 nM)	0.272 ± 0.018		
		ExTxA +Pn3a (1 µM)	0.068 ± 0.011		
		ExTxA +Pn3a + TTX (1 µM)	0.025 ± 0.008		
**Fig. 1k**	cumulative flinches (60 min) following i.pl. ExTxA (10 nM)	wt	319.3 ± 21.9	4	*p <0.0001 by two-tailed student's t-test
		$Na_V1.7^{Advill}$	40.0 ± 6.5	4	
**Fig. 2a,b**	Δ persistent current ($I_{40\ ms}/I_{peak}$ buffer *cf.* ExTxA)	HEK293-$Na_V1.1$	-0.003 ± 0.0040	11	
		HEK293-$Na_V1.2$	-0.002 ± 0.0053	11	

		HEK293-$Na_V1.3$	0.0265 ± 0.0049	11	*$p<0.0001$ by one-way ANOVA with Tukey's multiple comparison test
		HEK293-$Na_V1.4$	7.89E-07 ± 0.0005	24	
		HEK293-$Na_V1.5$	0.0004 ± 0.0005	9	
		HEK293-$Na_V1.6$	-0.008 ± 0.0103	12	
		HEK293-$Na_V1.7$	-0.0004 ± 0.0008	8	
		HEK293-$Na_V1.8$	0.027 ± 0.0057	11	
		ND7/23	-0.007 ± 0.0102	6	
		F11	0.0202 ± 0.0041	11	
		SH-SY5Y	0.006 ± 0.0175	6	
		h$Na_V1.7$ Xenopus oocytes	0.0381 ± 0.0074	5	
		TE-671	0.719 ± 0.0230	11	
**Fig. 2c,d**	Δ persistent current ($I_{40\ ms}/I_{peak}$) in CHO h$Na_V1.7$	no β subunit	0.001 ± 0.004	12	p > 0.05 by one-way ANOVA
		β1	0.0001 ± 0.001	11	
		β2	-0.002 ± 0.003	14	
		β3	-0.001 ± 0.002	15	
		β4	-0.004 ± 0.004	14	
**Fig. 3b**	Δ F/F in $Na_V1.7$ HEK293 (ExTxA 1 μM)	Control	0.016 ± 0.013	3	*p < 0.0001 by one-way ANOVA with Tukey's multiple comparison test
		Creld1	0.001 ± 0.0079	3	
		Lman2L	0.014 ± 0.0048	3	
		Mmgt1	0.0059 ± 0.0015	3	
		RNF121	0.007 ± 0.0074	3	
		Stt3b	0.013 ± 0.00098	3	
		GPI complex	0.0067 ± 0.0034	3	
		TMEM233	0.29 ± 0.0075	3	
**Fig. 3c**	ExTxA-induced persistent current ($I_{40\ ms}$; pA) in HEK293-h$Na_V1.7$	$Na_V1.7$ + ExTxA (1 μM)	-5.2 ± 3.7	11	*p < 0.0001 by two-tailed student's t-test
		$Na_V1.7$/ TMEM233 + ExTxA (1 μM)	1,473 ± 407.8	4	

**Fig. 3d**	ExTxA-induced persistent current ($I_{40\ ms}/I_{peak}$; nA) in TE-671 cells	Wildtype + ExTxA (1 µM)	0.81 ± 0.015	7	*p < 0.0001 by two-tailed student's t-test
		TMEM233 KO + ExTxA (1 µM)	0.047 ± 0.012	6	
**Fig. 3e**	ExTxA-induced persistent current (I_{40ms}/I_{peak}; nA) in DRG neurons	Wildtype + ExTxA (30 nM)	0.573 ± 0.086	6	*p < 0.0001 by two-way ANOVA
		Tmem233^{Cre} KO + ExTxA (30 nM)	0.114 ± 0.04	7	
**Fig. 3f**	ExTxA (10 nM)-induced DRG Ca^{2+} responses (% neurons activated)	wt	53.20 ± 1.789	10 wells	*p <0.0001 by two-tailed student's t-test
		Tmem233^{Cre} KO	22.33 ± 1.942	7 wells	
**Fig. 3g**	ExTxA (10 nM)-induced pain behaviours	wt	234.2 ± 27.7	17	*p = 0.037 by two-tailed student's t-test
		Tmem233^{Cre} KO	115.9 ± 25.3	16	
**Fig. 4a**	% positive signal (flow cytometry)	HEK293 + biotin-ExTxA (1 µM):	0.91 ± 0.51	3	*p < 0.05 (HEK293 vs. TMEM233, p = 0.002; HEK293 vs. $Na_V1.7$/TMEM233, p = 0.0015) by one-way ANOVA with Tukey's multiple comparison test
		TMEM233 + biotin-ExTxA (1 µM)	23.00 ± 2.66	3	
		$Na_V1.7$ + biotin-ExTxA (1 µM):	1.23 ± 0.28	3	
		$Na_V1.7$/TMEM233 + biotin-ExTxA (1 µM):	17.87 ± 2.93	3	
**Fig. 4b**	Max fluorescence (AFU)	HEK293 + Alexa488-ExTxA (1 µM):	15,740 ± 2133	3	

		TMEM233 + Alexa488-ExTxA (1 μM)	38,681 ± 951.2	3	*$p < 0.001$ by one-way ANOVA with Tukey's multiple comparison test
		$Na_V1.7$ + Alexa488-ExTxA (1 μM):	20,583 ± 3970	3	
		$Na_V1.7$/TMEM233 + Alexa488-ExTxA (1 μM):	38,799 ± 2471	3	
**Fig. 4d**	persistent current ($I_{40\ ms}/I_{peak}$)	$Na_V1.7$ + ExTxA (100 nM) in ECS	0.04 ± 0.014	10	*$p < 0.0001$ by two-way ANOVA with Šídák's multiple comparisons test
		$Na_V1.7$ + ExTxA (100 nM) in ICS	-0.002 ± 0.004	10	
		$Na_V1.7$/TMEM233 + ExTxA (100 nM) in ECS	0.543 ± 0.061	6	
		$Na_V1.7$/TMEM233 + ExTxA (100 nM) in ICS	0.003 ± 0.023	6	
**Fig. 4e,f**	% positive signal (flow cytometry)	C-terminal HA-tagged TMEM233, permeabilized	93.93 ± 3.526	3	*$p < 0.0001$ by one-way ANOVA with Tukey's multiple comparison test
		N-terminal HA-tagged TMEM233, permeabilized	93.17 ± 2.696	3	
		C-terminal HA-tagged TMEM233, surface	97.00 ± 0.1732	3	
		N-terminal HA-tagged TMEM233, surface	15.33 ± 1.534	3	
**Fig. 4g**	ExTxA EC_{50}	$Na_V1.7$/TMEM233	4.3 – 7.9 nM (95% CI)	7	$p < 0.05$ by one-way ANOVA with Tukey's multiple comparison test
		$Na_V1.7$/PRRT2	128.2 – 611.0 nM (95% CI)	10	
		$Na_V1.7$/TRARG1	308.6 – 6570.0 nM (95% CI)	7	

**Fig. 5e**	Proximity ligation assay (particles/cell)	HEK293 β1/β2 control	2.94 ± 0.21	3	*p < 0.0001 by one-way ANOVA with Tukey's multiple comparison test
		FLAG-tagged Na_V1.7	5.25 ± 1.68	3	
		N-terminal HA-tagged TMEM233	7.49 ± 1.82	3	
		FLAG-tagged Na_V1.7 + N-terminal HA-tagged TMEM233	36.72 ± 5.22	3	
**Fig. 6c,d,e**	V_{50} of activation (mV)	Na_V1.7	-26.11 ± 1.74	6	p > 0.05 by two-sided student's t-test
		Na_V1.7/TMEM233	-26.55 ± 1.40	11	
	V_{50} of inactivation (mV)	Na_V1.7	-64.95 ± 0.76	6	*p = 0.0045 by two-sided student's t-test
		Na_V1.7/TMEM233	-68.89 ± 0.76	11	
**Fig. 6f**	Slow inactivation best-fit parameters	Na_V1.7	95% CI<br>Bottom: 0.36 to 0.41<br>Top: 0.97 to 1.02<br>V_{50}: -60.99 to -57.13<br>Slope: -9.57 to -4.03	11	
		Na_V1.7/TMEM233	95% CI<br>Bottom: 0.19 to 0.26<br>Top: 0.99 to 1.07<br>V_{50}: -67.99 to -63.27<br>Slope: -9.11 to -5.16	13	
**Fig. 6g**	V_{50} of slow inactivation (mV)	Na_V1.7	-59.66 ± 0.61	11	*p = 0.0108 by two-tailed student's t-test
		Na_V1.7/TMEM233	-65.18 ± 1.74	13	

**Fig. 6h,i**	τ of fast inactivation	$Na_V1.7$	6.70 ± 0.75	8	*p = 0.0236 by two-tailed student's t-test
		$Na_V1.7$/TMEM233	9.64 ± 0.73	18	
**Fig. 6j**	Ramp currents (1 mV/ms) (nA)	$Na_V1.7$	-0.18 ± 0.04	11	p > 0.05 by two-tailed student's t-test
		$Na_V1.7$/TMEM233	-0.12 ± 0.01	9	
**Fig. 6l**	Use-dependence (I_{first}/I_{last} @ 20 mHz)	$Na_V1.7$	0.80 ± 0.03	8	*p = 0.016 by two-way ANOVA with Šídák's multiple comparisons test
		$Na_V1.7$/TMEM233	0.68 ± 0.07	6	
**Fig. 6m**	τ of fast inactivation	$Na_V1.7$	6.05 ± 0.52	16	*p < 0.05 by one-way ANOVA with Tukey's multiple comparison test
		$Na_V1.7$/TMEM233	10.53 ± 1.07	7	
		$Na_V1.7$/del.1-34 TMEM233	6.29 ± 0.49	7	
		Buffer control	6.91 ± 0.39	9	
		N-terminal TMEM233 peptide	10.18 ± 1.30	9	
**Supplementary figures**					
**Fig. 1e**	CHO cells persistent current ($I_{40\ ms}$, nA)	$hNa_V1.7$ + ExTxA (1 μM)	0.000 ± 0.002	6	*p = 0.0003 by two-tailed student's t-test
		$hNa_V1.7$/ TMEM233 + ExTxA (1 μM)	-2.010 ± 0.277	16	
**Fig. 1f**	*Xenopus oocyte* persistent current ($I_{40\ ms}/I_{peak}$)	$hNa_V1.7$ + ExTxA (1 μM)	0.081 ± 0.012	6	*p < 0.0001 by two-tailed student's t-test
		$hNa_V1.7$/ TMEM233 + ExTxA (1 μM)	0.727 ± 0.020	6	
**Fig. 1g**	Cos-1 cells Peak current (pA)	control	-284.6 ± 31.92	7	p >0.05 by two-tailed student's t-test
		TMEM233	-228.9 ± 14.15	6	
**Fig. 1h**	Cos-1 cells	Cos-1 + ExTxA (1 μM)	-2.019 ± 5.908	6	p >0.05 by two-tailed

	ExTxA (1 μM)-induced persistent current (pA)	Cos-1/TMEM233 + ExTxA (1 μM)	-7.493 ± 8.833	6	student's t-test
**Fig. 3c**	Best-fit parameters	ExTxA	Bottom: 0.0729<br>Top: 0.7692<br>$logEC_{50}$: -8.146<br>Hill slope: 1.156	7	
		Biotin-ExTxA	Bottom: -0.0014<br>Top: 0.7788<br>$logEC_{50}$: -6.256<br>Hill slope: 0.9303	6	
		Alexa488-ExTxA	Bottom: 0.00002<br>Top: 0.8013<br>$logEC_{50}$: -7.290<br>Hill slope: 1.084	7	
**Fig. 4b,c,d**	V_{50} of activation (mV)	$Na_V1.7$/TMEM233	-24.31 ± 1.11	7	*p < 0.0001 by two-tailed student's t-test
		$Na_V1.7$/TMEM233 + ExTxA (1 μM)	-35.92 ± 1.00	7	
**Fig. 4c,e**	V_{50} of inactivation (mV)	$Na_V1.7$/TMEM233	-67.77 ± 1.12	8	*p < 0.0001 by two-tailed Student's t-test
		$Na_V1.7$/TMEM233 + ExTxA (1 μM)	-49.80 ± 3.20	8	*p < 0.0001 by two-tailed student's t-test
**Fig. 4f**	τ_1 (recovery from fast inactivation)	$Na_V1.7$/TMEM233	7.23 ± 0.41	9	*p < 0.0001 by two-tailed student's t-test
		$Na_V1.7$/TMEM233 + ExTxA (1 μM)	1.17 ± 0.52	9	
**Fig. 4g**	Ramp current (0.2 mV/s) (nA)	$Na_V1.7$/TMEM233	-0.075 ± 0.012	9	*p < 0.0001 by two-tailed student's t-test
		$Na_V1.7$/TMEM233 + ExTxA (1 μM)	-1.952 ± 0.950	8	

**Fig. 5a**	V_{50} of activation (mV)	$Na_V1.7$	-17.53 ± 0.69	27	p > 0.05 by two-tailed student's t-test
		$Na_V1.7$/TMEM233	-17.38 ± 1.05	27	
	V_{50} of inactivation (mV)	$Na_V1.7$	-44.82 ± 0.54	22	p > 0.05 by two-tailed student's t-test
		$Na_V1.7$/TMEM233	-44.99 ± 0.72	24	
**Fig. 5b,c**	V_{50} of slow inactivation (mV)	$Na_V1.7$	-70.76 ± 0.54	22	p = 0.036 by two-tailed student's t-test
		$Na_V1.7$/TMEM233	-72.63 ± 0.66	24	
**Fig. 5d,e**	τ_1 (recovery from fast inactivation)	$Na_V1.7$	25.35 ± 1.89	21	*p = 0.0125 by two-tailed student's t-test
		$Na_V1.7$/TMEM233	39.01 ± 4.86	21	
