# Supplementary material for: Pain-causing stinging nettle toxins target TMEM233 to modulate NaV1.7 function
Source: Nat Commun. 2023 Apr 28;14:2442. doi: 10.1038/s41467-023-37963-2 (PMC10147923; doi:10.1038/s41467-023-37963-2)
Supplement: Supplementary file 3 — Reporting Summary [file 41467_2023_37963_MOESM3_ESM.pdf]

nature portfolio

Corresponding author(s): Irina Vetter, Thomas Durek

Last updated by author(s): Apr 4, 2023

Reporting Summary

Nature Portfolio wishes to improve the reproducibility of the work that we publish. This form provides structure for consistency and transparency in reporting. For further information on Nature Portfolio policies, see our Editorial Policies and the Editorial Policy Checklist.

Statistics

For all statistical analyses, confirm that the following items are present in the figure legend, table legend, main text, or Methods section.

n/a	Confirmed	
☐	☒	The exact sample size (*n*) for each experimental group/condition, given as a discrete number and unit of measurement
☐	☒	A statement on whether measurements were taken from distinct samples or whether the same sample was measured repeatedly
☐	☒	The statistical test(s) used AND whether they are one- or two-sided<br>*Only common tests should be described solely by name; describe more complex techniques in the Methods section.*
☒	☐	A description of all covariates tested
☐	☒	A description of any assumptions or corrections, such as tests of normality and adjustment for multiple comparisons
☐	☒	A full description of the statistical parameters including central tendency (e.g. means) or other basic estimates (e.g. regression coefficient) AND variation (e.g. standard deviation) or associated estimates of uncertainty (e.g. confidence intervals)
☐	☒	For null hypothesis testing, the test statistic (e.g. F, t, r) with confidence intervals, effect sizes, degrees of freedom and P value noted<br>*Give P values as exact values whenever suitable.*
☒	☐	For Bayesian analysis, information on the choice of priors and Markov chain Monte Carlo settings
☒	☐	For hierarchical and complex designs, identification of the appropriate level for tests and full reporting of outcomes
☒	☐	Estimates of effect sizes (e.g. Cohen's *d*, Pearson's *r*), indicating how they were calculated

Our web collection on statistics for biologists contains articles on many of the points above.

Software and code

Policy information about availability of computer code

Data collection	NMR spectra were processed using TOPSPIN v4.1.4 software (Bruker, Massachusetts, USA).<br>Automated electrophysiology was processed using Sophion Analyzer Version 6.6.64 (Sophion Bioscience, Ballerup, Denmark) and PatchMaster v2x90.4 beta software (HEKA Elektronik GmbH, Lambrecht/Pfalz, Germany).<br>Oocyte electrophysiology was processed using pCLAMP 11 software (Digidata 1550B; Axon Instruments).<br>DRG electrophysiology was processed using Clampex 10.7 (Molecular Devices), Clampfit 10.7 (Molecular Devices), Digidata 1440A (Molecular Devices), pClamp (Version 10).<br>Human iPSC electrophysiology was processed using Clampex 10.7 and Clampfit 10.7 (Molecular Devices).<br>sgRNA sequences in the TKOv3 screen were recovered using Checkout [http://100bp.wordpress.com] and cutadapt (ver.1.12).<br>FLIPR data was collected using ScreenWorks (Molecular Devices, Version 5.1.2.94).
Data analysis	Mass analysis was performed using Analyst QS Software 2.0 (Applied Biosystems, MDS SCIEX).<br>Enrichment of sgRNAs and genes were analyzed using MAGeCK (ver. 0.5.9).<br>NaV1.8-null and NaV1.9-null DRG electrophysiology data were plotted and analyzed using OriginPro 2021b (Microcal Software).<br>Proximity ligation assay signal (PLA particles) was quantified using ImageJ2 Version 2.9.0/1.53t.<br>RNAscope in situ Hybridization (ISH) images were edited in Adobe Lightroom v6 (Adobe).<br>Flow cytometry data were analysed using FlowJo™ (version 10.7.2, FlowJo, LLC, Ashland, OR, USA).<br>All other data were plotted and analyzed using GraphPad Prism Version 9.4.1.

For manuscripts utilizing custom algorithms or software that are central to the research but not yet described in published literature, software must be made available to editors and reviewers. We strongly encourage code deposition in a community repository (e.g. GitHub). See the Nature Portfolio guidelines for submitting code & software for further information.

Data

Policy information about availability of data

All manuscripts must include a data availability statement. This statement should provide the following information, where applicable:

- Accession codes, unique identifiers, or web links for publicly available datasets
- A description of any restrictions on data availability
- For clinical datasets or third party data, please ensure that the statement adheres to our policy

The data that support the findings of this study are available within the article, Supplementary Information, or Source Data file.
Sequences of the human dispanins were retrieved from UniProt: TMEM233 (DSPB2; UniProt ID B4DJY2), PRRT2 (DSPB3; UniProt ID Q7Z6L0), and TRARG1 (DSPB1; UniProt ID Q8IXB3).

Human research participants

Policy information about studies involving human research participants and Sex and Gender in Research.

Reporting on sex and gender	N/A
Population characteristics	N/A
Recruitment	N/A
Ethics oversight	N/A

Note that full information on the approval of the study protocol must also be provided in the manuscript.

Field-specific reporting

Please select the one below that is the best fit for your research. If you are not sure, read the appropriate sections before making your selection.

☒ Life sciences ☐ Behavioural & social sciences ☐ Ecological, evolutionary & environmental sciences

For a reference copy of the document with all sections, see nature.com/documents/nr-reporting-summary-flat.pdf

Life sciences study design

All studies must disclose on these points even when the disclosure is negative.

Sample size	Sample size calculations were done with an alpha of 0.05 for 80% power where appropriate.
Data exclusions	No data were excluded.
Replication	All data is presented with a minimum of 3 biological replicates. All replicates yielded similar results.
Randomization	Treatment randomization is not relevant to mouse in vivo experiments presented as all animals/genotypes received the same treatment. The order of genotypes assessed in behavioural assays was randomized. Different genotypes were interspersed and assessed together rather than in separate groups. For drosophila assays, animals were randomly allocated to treatment or control groups.<br>Randomization was not performed in in vitro experiment with pairwise design such as electrophysiological recordings pre- and post-toxin exposure.
Blinding	Experiments involving pain behaviors, consisting of paw licks or flinches, were counted from video recordings by a blinded investigator.<br>Blinding was not relevant to other in vitro experiments presented as measurements were analyzed by applying the same automated criteria.

Reporting for specific materials, systems and methods

We require information from authors about some types of materials, experimental systems and methods used in many studies. Here, indicate whether each material, system or method listed is relevant to your study. If you are not sure if a list item applies to your research, read the appropriate section before selecting a response.

Materials & experimental systems

n/a	Involved in the study
☐	☒ Antibodies
☐	☒ Eukaryotic cell lines
☒	☐ Palaeontology and archaeology
☐	☒ Animals and other organisms
☒	☐ Clinical data
☒	☐ Dual use research of concern

Methods

n/a	Involved in the study
☒	☐ ChIP-seq
☐	☒ Flow cytometry
☒	☐ MRI-based neuroimaging

Antibodies

Antibodies used

Proximity Ligation Assay:
mouse anti-FLAG antibody; Sigma F1804; 1:1000
rabbit anti-HA antibody; Sigma H6908; 1:1000
donkey anti-mouse PLUS; Sigma DUO92001; 1:5
donkey anti-rabbit MINUS; Sigma DUO92005; 1:5
Confocal microscopy:
mouse anti-HA (#26183, ThermoFisher Scientific); 1:1000
donkey anti-mouse antibody conjugated with alexafluor-488 (#715-545-150, Jackson ImmunoResearch Laboratories inc.); 1:1000

Validation

All antibodies were commercial and not validated in house.

Eukaryotic cell lines

Policy information about cell lines and Sex and Gender in Research

Cell line source(s)

TE-671 (European Collection of Cell Cultures, Porton Down, Salisbury, United Kingdom)
Human Embryonic Kidney (HEK) 293 cells (American Tissue Culture Collection, Manassas, VA, USA)
Stable HEK293-NaV1.1 (SB Drug Discovery, Glasgow, United Kingdom)
Stable HEK293-NaV1.2 (SB Drug Discovery, Glasgow, United Kingdom)
Stable HEK293-NaV1.3 (SB Drug Discovery, Glasgow, United Kingdom)
Stable HEK293-NaV1.4 (SB Drug Discovery, Glasgow, United Kingdom)
Stable HEK293-NaV1.5 (SB Drug Discovery, Glasgow, United Kingdom)
Stable HEK293-NaV1.6 (SB Drug Discovery, Glasgow, United Kingdom)
Stable HEK293-NaV1.7 (SB Drug Discovery, Glasgow, United Kingdom)
Stable CHO-NaV1.7 (ChanTest, Ohio, USA)
Stable CHO-NaV1.8 (ChanTest, Ohio, USA)
ND7/23 (Sigma Aldrich Australia)
F11 (Sigma Aldrich Australia, European Cell Culture Collection)
SH-SY5Y (Sigma Aldrich Australia, European Cell Culture Collection)
Human iPSC-derived sensory neurons (RealDRG™) produced by Anatomic Inc. from the female subject ANAT001

Authentication

Cell lines were not authenticated.

Mycoplasma contamination

Cell lines were not tested for mycoplasma contamination

Commonly misidentified lines
(See ICLAC register)

TE-671 cells were originally identified as medulloblastoma but have since been reassigned as rhabdomyosarcoma. This has no impact on the study as the cells were used to identify the pharmacological target of ExTxA.

Animals and other research organisms

Policy information about studies involving animals; ARRIVE guidelines recommended for reporting animal research, and Sex and Gender in Research

Laboratory animals

C57BL/6 - Animal Resource Centre (Canning Vale, Western Australia, Australia) or local breeding colonies.
TMEM233Cre/Cre on C57BL/6 background - see Santana-Varela, S. et al. Wellcome Open Res 2021.
NaV1.7flox x Advillin-Cre on C57BL/6 background - see Minett, M. S. et al. Nat Commun 2012.
NaV1.8Cre/Cre on C57BL/6 background - see Nassar, M. A. et al. Proc Natl Acad Sci 2004
NaV1.9-/- mice on C57BL/6 background - see Amaya, F. et al. J Neurosci 2006
CAG-floxed stop tdTomato on C57BL/6 background (Jackson Laboratory, Maine, USA; strain 007914)
Mice used in the study were aged 1-12 months.
Mice were housed in groups of three or four per cage under 12-hour light-dark cycles and had standard rodent chow and water ad libitum.
Oocytes were isolated from adult female Xenopus laevis - Xenopus express (Florida, United states)
Oocytes used in the study were stage V/VI
Drosophila melanogaster, aged 3 days

Wild animals	The study did not involve wild animals
Reporting on sex	Both male and female mice were used in this study. The study was not designed or powered to detect sex specific differences. In the insecticidal assay female Drosophila melanogaster were used to minimize weight variability.
Field-collected samples	The study did not involve samples collected from the field
Ethics oversight	All experiments involving animals were reviewed and approved by local Institutional animal ethics committees (US Veterans Affairs West Haven Medical Center Animal Care and Use Committee; University of Queensland Molecular Bioscience Animal Ethics Committee; King's College London Animal Welfare and Ethical Review Body) and conducted in accordance with relevant national and international regulations (International Association for the Study of Pain Guidelines for the Study of Pain Guidelines for the Use of Animals in Research; the Australian Code of Practice for the Care and Use of Animals for Scientific Purposes, 8th edition (2013); UK Home Office Project License). X. laevis oocyte surgeries were reviewed and approved by the Anatomical Biosciences group of the Animal Ethics Committee at The University of Queensland (QBI/AIBN/087/16/NHMRC/ARC) and conducted in accordance with Australian quarantine regulations.

Note that full information on the approval of the study protocol must also be provided in the manuscript.

Flow Cytometry

Plots

Confirm that:

☒ The axis labels state the marker and fluorochrome used (e.g. CD4-FITC).

☒ The axis scales are clearly visible. Include numbers along axes only for bottom left plot of group (a 'group' is an analysis of identical markers).

☒ All plots are contour plots with outliers or pseudocolor plots.

☒ A numerical value for number of cells or percentage (with statistics) is provided.

Methodology

Sample preparation	HEK293 cells (untransfected controls or transfected with TMEM233) as well as control and TMEM233-transfected HEK293-NaV1.7 cells were harvested using TrypLE Express, washed and resuspended in phosphate buffer solution. Cells were then pelleted (400 x g for 5 min) and re-suspended in physiological salt solution (PSS) containing 0.1% bovine serum albumin. Biotinylated ExTxA (1 µM) was then added to the cells and incubated on a shaker for 30 mins. Subsequently, cells were pelleted and washed three times with PSS. Cells were then incubated with Streptavidin DyLight 680 (1:2000) for 45 mins with agitation. Following, cells were washed three times with PSS and resuspended in PSS.
Instrument	BD FACSAria Cell Sorter
Software	FlowJo™ (version 10.7.2, FlowJo, LLC, Ashland, OR, USA)
Cell population abundance	HEK293 cells were not sorted based on expression of TMEM233 and/or Nav1.7.
Gating strategy	All gates were created on the unstained control and then applied to the other samples. FSC-A vs. SSC-A gating was used to select the cell population and to eliminate cell debris. Afterwards, doublet discrimination was performed using SSC-H vs. SSC-W and FSC-H vs. FSC-W gating. In the FSC-A vs. APC-A dot plot, a threshold was set at the fluorescent intensity of 1,000 above which the events were considered as positive signal.

☒ Tick this box to confirm that a figure exemplifying the gating strategy is provided in the Supplementary Information.
